# *PTEN* and novel *TEK* germline variants associated with the phenotypes of *PTEN* hamartoma tumor syndrome

**DOI:** 10.1016/j.gendis.2024.101466

**Published:** 2024-11-16

**Authors:** Hongrui Chen, Bin Sun, Yanchun Zhou, Chen Hua, Xiaoxi Lin

**Affiliations:** Department of Plastic & Reconstructive Surgery, Shanghai Ninth People’s Hospital, Shanghai Jiao Tong University School of Medicine, Shanghai 200011, China

Phosphatase and tensin homolog (*PTEN*) is a tumor suppressor gene located at 10q23.3 and serves as a crucial negative regulator of the PI3K-AKT signaling cascade by dephosphorylating phosphatidylinositol (3,4,5)-triphosphate and inhibiting AKT activation. *PTEN* hamartoma tumor syndrome (PHTS) is an umbrella term employed to describe a spectrum of disorders caused by germline PTEN variants, including Cowden syndrome and Bannayan-Riley-Ruvalcaba syndrome. While distinct entitles included in PHTS have their unique diagnostic criteria, they also share common phenotypic features such as benign hamartomas of the three germ layers, macrocephaly, and an increased predisposition to malignancies in specific organs.^1^

Here, we describe a unique case harboring germline *PTEN* and *TEK* variants. His phenotypes include macrocephaly, scoliosis, adipose tissue hyperproliferation on the left side of the body, and vascular malformations on the right side. To our knowledge, this is the first case that describes the possible synergic role of coexisting variant of both *PTEN* and *TEK* in the development of PHTS, underlining the importance of tailored therapeutic management in these patients, especially during childhood.

A four-year-old boy visited our clinic with his parents. He was born at 38 weeks of gestation with no reported anomalies at birth. At age three, three more soft masses were found in his left inguinal area and back (Fig. 1A, B). He underwent an operation on the masses in his back in another hospital. The mass beneath his left scapula was excised, and a local incisional biopsy was performed on the lesion on his right waist. Pathologic results suggested that the mass beneath his left scapula was a lipoma. For the right lumbar mass, proliferative and dilated vessels were observed in the fibrous tissue. The immunohistochemical results were partly positive for D2-40 and PROX-1, and positive for CD31 and P-S6-235, consistent with lymphatic-venous malformation. The parents only carried the reports, so these pathological images are not displayed.

**Figure 1:**
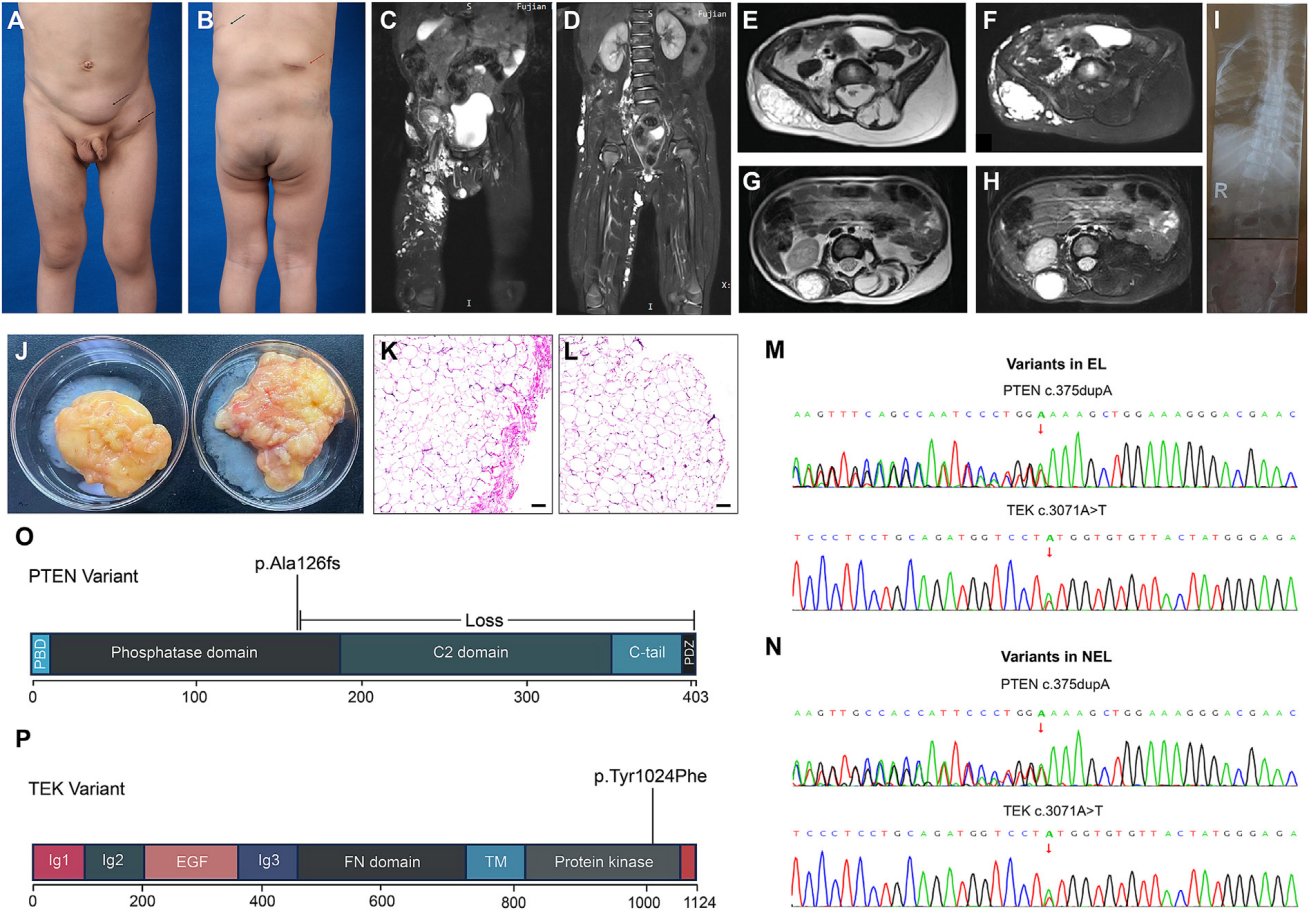
The patient’s phenotypic and genetic features. **(A)** Anterior view of the patient. The mass in his left lower abdomen and left inguinal region was excised (black arrow). **(B)** Posterior view of the patient. A mass below his left scapula was excised (black arrow). A biopsy of the swelling in the right lumbar region was performed in an outside hospital, suggesting a venous-lymphatic malformation (red arrow). **(C, D)** T2 coronal fat-suppressed images exhibited multiple high-signal lesions in various sizes persisting in the right groin, perineum, pelvis, middle and lower abdominal wall, lumbar dorsal region, buttock, and right thigh. Certain lesions showed substantial irregular enhancement (in the right lumbar dorsal region, right buttock, and right groin). **(E, F)** T2 axial and fat-suppressed images revealed a marked increase in subcutaneous fat, manifesting as a lump-like alteration anterior to the erector spinae and posterior to the left lamina at levels L4-S1. A lesion similar to a lymphatic malformation could be observed in the subcutaneous area of his right dorsal region. **(G, H)** T2 axial and fat-suppressed images presented a vascular malformation lesion situated behind his right kidney. **(I)** X-ray showed scoliosis. **(J)** The encapsulated lipoma (EL) located in the left inguinal region (left) and the non-encapsulated lipoma (NEL) in the left lower abdomen (right). **(K, L)** Hematoxylin and eosin staining of EL and NEL. A fibrous membrane could be observed overlying the EL. Scale bar, 200 μm. **(M, N)** Validation of PTEN and TEK variants in EL and NEL by Sanger sequencing. **(O)** Schematic representation of the PTEN domains. PTEN c.375dupA (p.Ala126fs) is a frameshift variant that can lead to the formation of a truncated protein composed of 126 amino acids or cause the loss of normal PTEN protein function through nonsense-mediated mRNA decay. **(P)** Schematic representation of the TEK domains. TEK c.3071A > T (p.Tyr1024Phe) is located in a critical functional domain with known benign variants.

When he presented to our department, we noticed that his head circumference was 57 cm (>97th percentile). We did not observe the macular pigmentation on the glans penis often seen in Bannayan-Riley-Ruvalcaba syndrome. Magnetic resonance imaging revealed a symmetric enlargement of the bilateral cerebral hemispheres (megalencephaly), with no other detectable neurological abnormalities. Based on our outpatient observations and the parents' reports, the boy did not exhibit developmental delay or impairments in language, cognition, or emotional development.

Multiple size-variable lesions in the soft tissues of the right body were noticed, which appear to be consistent with the characteristics of venous malformations (Fig. 1C, D). Notable fat accumulation was observed on his left back, particularly around the L4–S1 spine level (Fig. 1E–H). X-ray imaging revealed scoliosis (Fig. 1I). Further surgical excision was performed on two lipomas in his left lower abdomen which appeared to be encapsulated and non-encapsulated respectively (Fig. 1J). Histological examination confirmed this observation (Fig. 1K, L). Due to a suspected genetic etiology for his multifocal lipomas, genetic analysis was done, identifying heterozygous pathogenic *PTEN* variant (exon 5; c.375dupA; p.Ala126fs; NM_000314) and *TEK* variant (exon20; c.3071A > T p.Tyr1024Phe; NM_000459) (Fig. 1O, P). These results were validated by Sanger sequencing (Fig. 1M, N). Genetic testing of peripheral blood revealed identical *PTEN* and *TEK* variants, with variant allele frequencies of 46.3% and 52.4% respectively, confirming them as germline variants and further clarifying the diagnosis of PHTS. Since parental testing could not be pursued, we do not have any information about the inheritance of these variants. However, his parents did not present any PHTS-related lesions. No abnormalities were detected in the thyroid ultrasound. Considering his age and the fact that he does not currently have any digestive symptoms, we did not perform a colonoscopy. He continues to be monitored with plans for further surgery as he grows up.

The diagnostic criteria for PHTS have been thoroughly described.^1^ This case meets one major criterion (macrocephaly) and two minor criteria (vascular malformation and multiple lipomas). The typical manifestations of Cowden syndrome include an increased risk of malignancies, such as breast, thyroid, and endometrial cancers, as well as benign hamartomatous overgrowth in tissues. Meanwhile, Bannayan-Riley-Ruvalcaba syndrome is distinguished by features such as macrocephaly, hamartomatous intestinal polyps, lipomas, and pigmented macules on the penis. A previous report highlighted a Bannayan-Riley-Ruvalcaba syndrome case where penile freckles, not present at birth, became evident when the patient reached the age of nine. Due to the patient’s young age, related pathologies may not have yet manifested.

Morgan et al reported a patient with phenotypes including macrocephaly, gastrointestinal polyps, and arteriovenous malformations, who was found to have a *PTEN* (c.388C > T; p.R130∗; NM_000314.4) germline variant. Yet genetic testing was not conducted on her parents. Gordian and colleagues described another case of a child carrying a *PTEN* germline deletion variant, with both the parents and siblings testing negative.^2^ Neither of these cases explained the inheritance of the *PTEN* germline variations. A study revealed that the frequency of *de novo* germline variants in PHTS ranged from 10.7% to 47.6%.^3^ Since his parents did not have any PHTS-related manifestations, we hypothesized that this patient might have a *de novo* variant.

Additional variants may contribute to further clinical phenotypes in PHTS. Gaia et al reported a case of a Bannayan-Riley-Ruvalcaba syndrome patient who harbored both *PTEN* and *TPO* variants. The concurrent *TPO* variant resulted in the patient developing a multinodular goiter.^4^ Arteriovenous malformations are more prevalent in PHTS. Conversely, venous malformations appear to be less frequent. It is plausible to theorize that the concurrent *TEK* variant may contribute to the progression of extensive venous malformations in our patient. *TEK* is located at 9p21.2 and encodes for endothelial cell tyrosine kinase receptor TIE2 which is involved in the regulation of angiogenesis. Signals from TIE2 stimulate the activation of the PI3K-AKT signaling pathway. Gain-of-function variants in *TEK* could provide an alternative stimulus for AKT activation,^5^ which suggests that the concurrent presence of *TEK* variants might exacerbate the phenotypic manifestations associated with *PTEN* variants by further promoting AKT pathway activation. Notably, the *TEK* p.Tyr1024Phe variant identified in our patient has been classified as ACMG tier III (a variant of uncertain significance). Tyr1024 is a highly conserved residue within the activation loop of the kinase domain, and its substitution for phenylalanine could potentially alter the kinase’s conformation and activity. However, the precise impact of this variant on *TEK* function — whether it results in a gain, loss, or neutral effect — requires further experimental validation.

While surgical intervention could address the patient’s multiple lipomas and the hyperproliferation of the subcutaneous fat, achieving a complete eradication of the lesions proved challenging. This spurred our pursuit of targeted, etiology-based therapeutic strategies. Given that the dysfunction of *PTEN* can lead to the overactivation of mTOR, sirolimus has been reported for application in PHTS, achieving local or systemic therapeutic effects. Sirolimus demonstrates notable efficacy for vascular malformations, yet its inhibitory effect on adipose tissue seems limited. Recently, the PI3Kα inhibitor alpelisib has been approved for the treatment of overgrowth diseases caused by *PIK3CA* variants, exhibiting efficacy for the overgrowth of various mesoderm-derived components (fat, bone, muscle, blood vessels, *etc*.). Currently, alpelisib is undergoing relevant clinical trials in China. Given the shared pathogenic signaling pathway, it is reasonable to anticipate potential therapeutic prospects of alpelisib for PHTS.

## Ethics declaration

All procedures were conducted based on the ethics approval obtained from the Ethics Board of Shanghai Ninth Hospital, Shanghai Jiao Tong University of Medicine (No. SH9H-2021-C46). Diagnostic genetic testing was performed after written informed consent was obtained from the patient’s parents and in accordance with local regulations. The patient’s parents gave their consent for the photographs and medical information to be published in print and online with the understanding that this information may be publicly available.

## Funding

This work was supported by the Major and Key Cultivation Projects of Ninth People’s Hospital affiliated to Shanghai Jiao Tong University School of Medicine (No. JYZP005), the Top Priority Research Center of Shanghai-Plastic Surgery Research Center, China (No. 2023ZZ02023), the Fundamental Research Funds for the Central Universities of China (No. YG2023ZD13), and the Treatment and Mechanism of PI3K/mTOR Dual-Target Inhibitor (WX390) on PIK3CA-Related Overgrowth Spectrum (PROS) (China) (No. JYHX2022004).

## CRediT authorship contribution statement

**Hongrui Chen:** Writing – original draft. **Bin Sun:** Writing – original draft. **Yanchun Zhou:** Investigation, Resources, Software, Supervision. **Chen Hua:** Writing – review & editing. **Xiaoxi Lin:** Writing – review & editing.

## Conflict of interests

The authors have no conflict of interests relevant to this article to disclose.

## References

[1] Pilarski R., Burt R., Kohlman W., Pho L., Shannon K.M., Swisher E. (2013). Cowden syndrome and the PTEN hamartoma tumor syndrome: systematic review and revised diagnostic criteria. J Natl Cancer Inst.

[2] Dykman M., Pillai N.R., Lenhart K. (2024). Arteriovenous malformations as a presenting sign of PTEN hamartoma tumor syndrome: a case series. Pediatr Dermatol.

[3] Mester J., Eng C. (2012). Estimate of *de novo* mutation frequency in probands with PTEN hamartoma tumor syndrome. Genet Med.

[4] Vincenzi G., Petralia I.T., Abbate M. (2023). Case report - multinodular goiter in a patient with congenital hypothyroidism and Bannayan-Riley-Ruvalcaba syndrome: the possible synergic role of TPO and PTEN mutation. Front Endocrinol.

[5] Du Z., Zheng J., Zhang Z., Wang Y. (2017). Review of the endothelial pathogenic mechanism of TIE2-related venous malformation. J Vasc Surg Venous Lymphat Disord.

