# Supplementary material for: PTEN and novel TEK germline variants associated with the phenotypes of PTEN hamartoma tumor syndrome
Source: Genes Dis. 2024 Nov 16;12(4):101466. doi: 10.1016/j.gendis.2024.101466 (PMC11995074; doi:10.1016/j.gendis.2024.101466)
Supplement: Multimedia component 1 [file mmc1.docx]

***PTEN* and novel *TEK* germline variants associated with the phenotypes of *PTEN* hamartoma tumor syndrome**

**Materials & Methods**

**Imaging modality**

MR examinations were performed using a MAGNETOM Verio with a magnetic field strength of 3.0 Tesla. The imaging protocol for MR included axial and coronal T1-weighted images (T1WI), axial and coronal T2-weighted images (T2WI), and axial and coronal T1-weighted images with fat suppression post-contrast.

**Histopathological staining**

The samples were fixed in 10% (v/v) formalin, followed by dehydration in a series of gradient solutions. The fixed samples were then dehydrated in a gradient alcohol series. The tissue was washed in xylene and embedded in paraffin. The samples were sliced into sections approximately 5 μm thick and stained with haematoxylin and eosin.

**DNA extraction and next-generation sequencing (NGS)**

We performed targeted sequencing using a high-depth NGS approach. The DNA was extracted from cells using the Qiagen DNA Extraction Kit (Qiagen, #13323). Genomic DNA fragments were spliced and modified for sequencing with the NEBNextillinautraII DNA Library Preparation Kit. After library establishment was completed, high-throughput sequencing was performed using the Illumina Nova Seq 6000 platform. The NGS panel had an average sequencing depth of 10,000X and 98% coverage. DNA sequences from the assay samples were compared to the reference sequence hg19 (GRCh37) and analyzed to determine the possible mutations.

**Sanger sequencing**

Conventional PCR was performed using a Veriti thermocycler (Applied Biosystems, Thermo Fisher Scientific MA). Amplified PCR products were analyzed by Tsingke (Shanghai, China) for Sanger sequencing. The primers for PCR were as follows:

| Gene | Primer sequence |
| --- | --- |
| PTEN | F: TTGTATGCAACATTTCTAAAGTTACC |
|  | R: AATTCTCAGATCCAGGAAGAGGA |
| TEK | F: GCTAAGAGGAGAGAAACCTACACTG |
|  | R: AGAACCACATACAAACGGCATC |
